# Supplementary material for: Comparison of Associations between MIND and Mediterranean Diet Scores with Patient-Reported Outcomes in Parkinson’s Disease
Source: Nutrients. 2022 Dec 6;14(23):5185. doi: 10.3390/nu14235185 (PMC9739738; doi:10.3390/nu14235185)
Supplement: Supplementary file 1 [file nutrients-14-05185-s001.zip › nutrients-1984673-supplementary.pdf]

Table S1. Patient-Reported Outcomes in Parkinson's Disease (PRO-PD) scoring items. PRO-PD symptoms inclusion for total scale (left), non-motor symptoms (middle), and motor symptoms (right).

Total Pro-PD	Non-motor PRO-PD	Motor PRO-PD
Slowness	Constipation	Handwriting or Typing
Tremor	Motivation/initiative	Slowness
Balance	Depression	Tremor
Fatigue	Withdrawn	Stooped posture
Sleepy	Anxiety	Difficulty walking
Motivation/initiative	Fatigue	Difficulty speaking
Constipation	Daytime sleepiness	Rising from seated position
Walking	Visual disturbance	Dressing
Rising from seated position	Insomnia	Restless legs
Dressing	REM Sleep Behavior Disorder	Falling
Freezing	Muscle pain	Balance
Falling	Drooling	Freezing
Handwriting or Typing	Memory/forgetfulness	
Drooling	Comprehension	
Speech	Sexual dysfunction	
Visual disturbance	Urinary symptoms	
Muscle pain	Hallucinations	
Restless legs		
REM Sleep Behavior Disorder		
Insomnia		
Smell		
Nausea		
Depression		
Anxiety		
Withdrawn		
Dizziness		
Stoop posture		
Memory/forgetfulness		
Comprehension		
Sexual dysfunction		
Urinary symptoms		
Dyskinesia		
Hallucinations		

Table S2: Mediterranean Diet questionnaire on dietary habits and consumption frequency

Questionnaire Items	Criteria for 1 point
Do you use olive oil as a main culinary fat?	Primary cooking fat
Do you consume more than 4 tablespoons of olive oil in a given day	≥ 4 Tbs
Do you consume more than 2 vegetable servings per day, with at least one of the portions raw or as a salad?	> 2 servings/day with ≥ 1 raw
Do you consume 3 or more fruit units per day?	≥ 3 servings/day
Do you consume less than 1 serving per day of red meat, hamburger, or meat products?	<1 Serving/day
Do you consume less than 1 serving per day of butter, margarine, or cream?	<1 Serving/day
Do you consume less than 1 sweet or carbonated beverage drink per day?	<1 beverage/day
Do you drink 7 or more glasses of wine per week?	>7 glasses/week
Do you consume 3 or more servings of legumes per week?	≥3 servings/week
Do you consume 3 or more servings of fish or shellfish per week?	≥3 servings/week
Do you consume less than 3 servings per week of commercial sweets or pastries (not homemade), such as cakes, cookies, biscuits, or custard?	<3 servings/week
Do you consume 3 or more servings of nuts per week?	≥ 3 servings/week
Do you preferentially consume chicken, turkey, or rabbit meat?	Yes
Do you consume vegetables, pasta, rice, or other dishes seasoned with sofrito at least 2 times per week?	≥ 2 servings/week

Table S3: Mediterranean-DASH Intervention for Neurodegenerative Delay (MIND) diet components from the Food Frequency Questionnaire

**Mediterranean-DASH Intervention for Neurodegenerative Delay (MIND) Diet Scoring System**			
	**Points Assigned**		
**Questionnaire Items**	0	0.5	1
Green Leafy Vegetables	≤ 2 servings/week	3 to 5 servings/week	≥ 6 serving/week
Other Vegetables	≤ 4 servings/week	4 to 6 servings/week	≥ 1 serving/day
Berries	< 1 serving/week	1 serving/week	≥ 2 serving
Nuts	Less than once a month	1 serving/month to 4 servings/week	≥ 5 servings/week
Olive Oil	Not a primary oil	-	Is the primary oil
Butter, Margarine	≥ 2 Tbs /day	1 to 2 Tbs /day	≤ 1 Tbs /day
Cheese	≥7 servings/week	1 to 6 servings/week	<1 serving/week
Whole Grains	≤ 1 serving/week	1 to 2 servings/day	≥3 servings/week
Fish (not fried)	Rarely	1 to 3 meals/month	≥ 1 meal/week
Beans	< 1 meal /week	1 to 3 meals/week	≥ 4 meals/week
Poultry (not fried)	< 1 meal/week	1 meal/week	≥ 2 meals/week
Red Meat and Products	> 6 meals/week	4 to 6 meals/week	≤ 3 meals/week
Fast Fried Foods	≥ 4 meals/week	1 to 3 meals/week	<1 meal/week
Pastries and Sweets	≥ 7 times/week	5 to 6 times/week	≤ 4 times/week
Wine	Never	1/month to 6/week	1 glass/day
Total Score	0	7.5	15

Table S4: Individual items on MIND and Mediterranean Questionnaires and association with Patient Reported Outcomes in Parkinson's Disease symptom severity by linear regressions adjusted for age, gender, income, and years since diagnosis. Reverse coded items were adjusted to reflect higher consumption.

**The Relationship Between Individual Questions on the MIND and Mediterranean Questionnaires: Associations with Patient-Reported Parkinson Disease Symptoms Over Time**			
**Mediterranean Diet Adherence Questionnaire**	**Mean Score**	**Estimated Impact of Question on overall PRO-PD**	**p-value**
Do you consume less than 1 sweet or carbonated beverage drink per day?	0.80	-135 (-203- -68.2)	<0.001
Do you consume 3 or more servings of nuts (including peanuts) per week?	0.68	-102 (-161 - -43.6)	0.005
Do you consume less than 1 serving per day of red meat, hamburger, or meat products?	0.76	-95 (-156 - -28.3)	0.04
Do you consume more than 2 vegetable servings per day, with at least one of the portions raw or as a salad?	0.70	-95 (-156- -35.5)	0.02
Do you consume less than 1 serving per day of butter, margarine, or cream?	0.70	-67.7 (-127 - -8.57)	0.1
Do you consume les than 3 servings per week of commercial sweets or pastries (not homemade), such as cakes, cookies, biscuits, or custard?	0.55	-54.0 (-109 - 0.442)	0.3
Do you consume 3 or more fruit units (including natural fruit juices) per day?	0.60	-47.9 (-103 - 7.68)	0.5
Do you use olive oil as a main culinary fat?	0.77	-46.0 (-112 - 19.7)	0.7
Do you consume vegetables, pasta, rice, or other dishes seasoned with sofrito at least 2 times per week?	0.38	45.0 (-11.6 -102)	0.5
Do you consume 3 or more servings of legumes per week?	0.50	-43.6 (-98.2 - 11.0)	0.6
Do you drink 7 or more glasses of wine per week?	0.17	-41 (-114 - 32.6)	0.9
Do you consume 3 or more servings of fish or shellfish per week?	0.35	-31.0 (-88.9 - 27.0)	0.9
Do you preferentially consume chicken, turkey, or rabbit meat instead of veal, pork, hamburger, or sausage?	0.64	-27.7 (-85.3 - 29.9)	0.3
Do you consume more than 4 tablespoons of olive oil in a given day?	0.18	-7.65 (-79.3 - 64.0)	0.9
**Total Medi Score**	7.8	-25.6 (-37.2- -14)	<0.001
**MIND Dietary Adherence Questionnaire**			
How often do you consume nuts?	0.67	-198 (-276 - -120)	<0.001
How often do you consume green leafy vegetables?	0.51	-196 (-273 - -118)	<0.001
How often do you consume other vegetables?	0.57	-190 (-262 - -119)	<0.001
How often do you consume berries?	0.67	-119 (-184 - -54.4)	0.03
How often do you consume beans?	0.34	-111(-195- -27.8)	0.07
How often do you consume wine?	0.43	-99.5 (-175- -23.6)	0.08
How often do you consume non-fried fish?	0.61	-93.6 (-164- -23.6)	0.08
How often do you consume whole grains?	0.53	-71.7 (-137 - -6.80)	0.2
How often do you consume olive oil?	0.76	-52.7 (-117 - 11.7)	0.6
How often do you consume non-fried poultry?	0.61	60.1 (-5.33 - 125)	0.4
How often do you consume pastries and sweets	0.78	114 (195- 32.8)	0.06
How often do you consume fast fried food?	0.94	211 (263- 59.1)	0.06
How often do you consume cheese?	0.65	246 (338 - 154)	<0.001
How often do you consume red meat and related products?	0.90	254 (379- 130)	<0.001
How often do you consume butter or margarine?	0.84	273 (370 - 177)	<0.001
**Total MIND Score**	9.8	-52 (-66.4 - -39.4)	<0.001

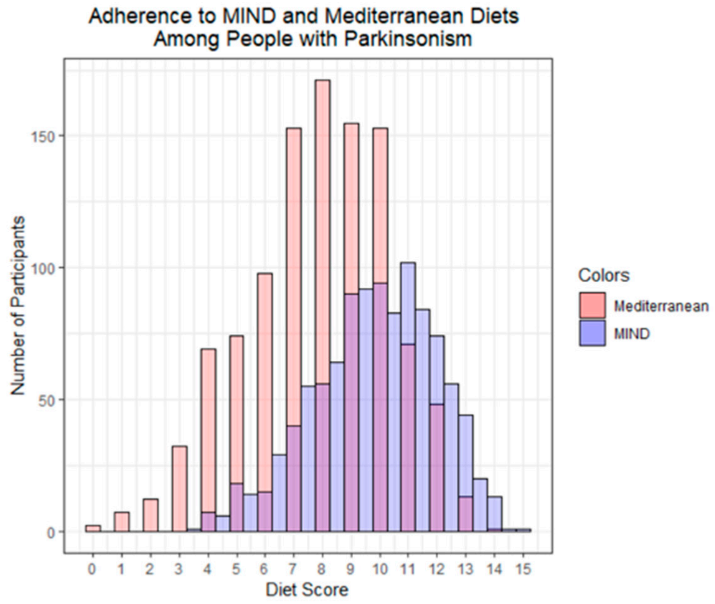

Figure S1: Mediterranean and MIND diet score distribution amoung individuals with Parkinsonism
